# Iron Metabolism: An Under Investigated Driver of Renal Pathology in Lupus Nephritis

**DOI:** 10.3389/fmed.2021.643686

**Published:** 2021-04-12

**Authors:** Ewa Wlazlo, Borna Mehrad, Laurence Morel, Yogesh Scindia

**Affiliations:** ^1^Department of Medicine, University of Virginia School of Medicine, Charlottesville, VA, United States; ^2^Division of Pulmonary, Critical Care, and Sleep Medicine, University of Florida, Gainesville, FL, United States; ^3^Department of Pathology, University of Florida, Gainesville, FL, United States; ^4^Division of Nephrology, University of Florida, Gainesville, FL, United States

**Keywords:** iron, proximal renal tubular cells, lupus nephritis, glomerulonephritis, SLE, ferroptosis

## Abstract

Nephritis is a common manifestation of systemic lupus erythematosus, a condition associated with inflammation and iron imbalance. Renal tubules are the work horse of the nephron. They contain a large number of mitochondria that require iron for oxidative phosphorylation, and a tight control of intracellular iron prevents excessive generation of reactive oxygen species. Iron supply to the kidney is dependent on systemic iron availability, which is regulated by the hepcidin-ferroportin axis. Most of the filtered plasma iron is reabsorbed in proximal tubules, a process that is controlled in part by iron regulatory proteins. This review summarizes tubulointerstitial injury in lupus nephritis and current understanding of how renal tubular cells regulate intracellular iron levels, highlighting the role of iron imbalance in the proximal tubules as a driver of tubulointerstitial injury in lupus nephritis. We propose a model based on the dynamic ability of iron to catalyze reactive oxygen species, which can lead to an accumulation of lipid hydroperoxides in proximal tubular epithelial cells. These iron-catalyzed oxidative species can also accentuate protein and autoantibody-induced inflammatory transcription factors leading to matrix, cytokine/chemokine production and immune cell infiltration. This could potentially explain the interplay between increased glomerular permeability and the ensuing tubular injury, tubulointerstitial inflammation and progression to renal failure in LN, and open new avenues of research to develop novel therapies targeting iron metabolism.

## Lupus Nephritis: A Brief Background

**Figure 1:**
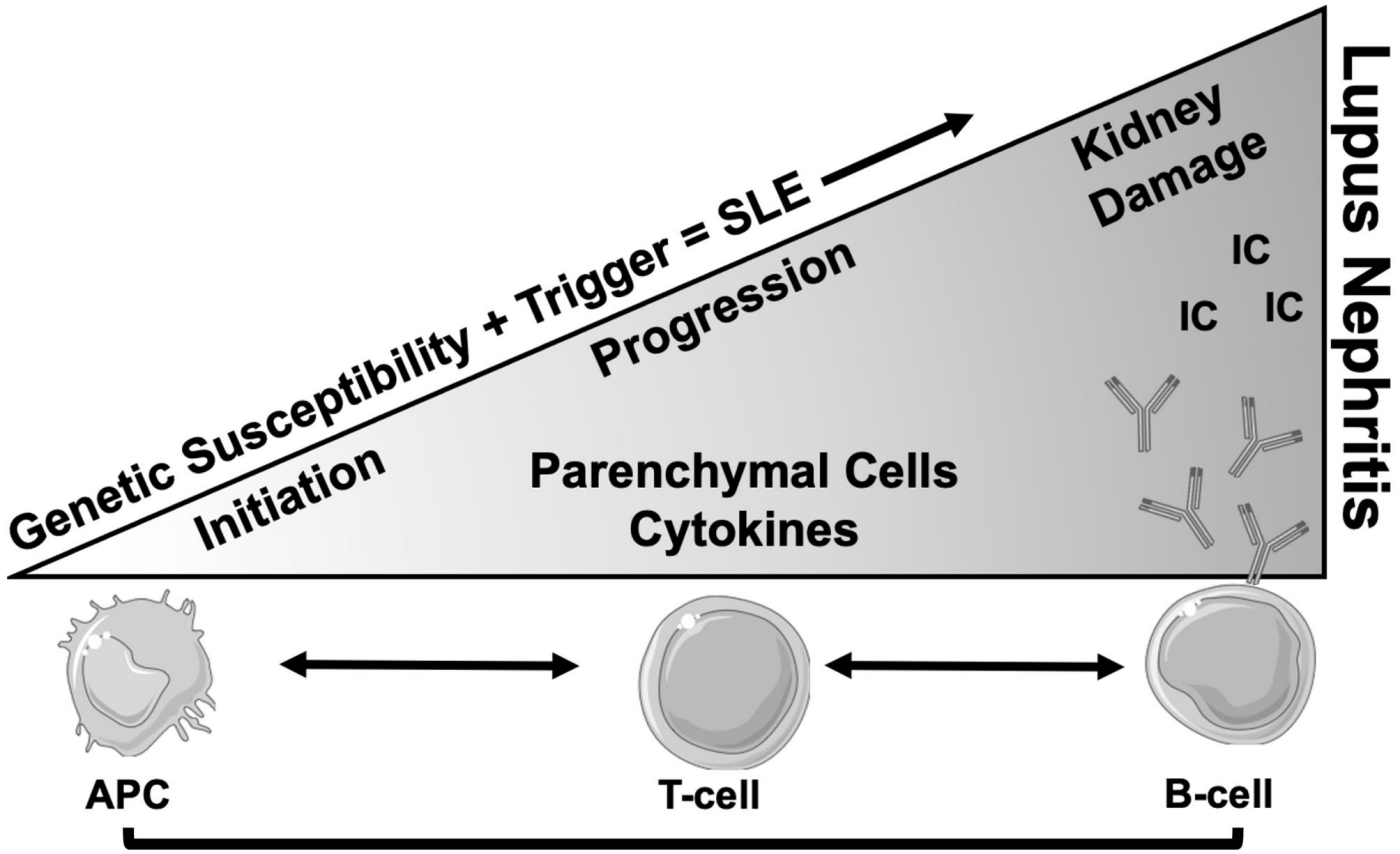
Schema of progression of SLE to end organ renal disease (LN). After the onset of SLE in a genetically susceptible individual, there is involvement of multiple cells types involving both innate and adaptive immune systems. The antigen presenting cells (APC) present self-antigens from various sources to T lymphocytes, which results in generation of auto reactive T cells with low activation threshold. These CD4 T lymphocytes in turn instruct B cells to produce autoantibodies (Y) of different specificities. Not much is known about the cause and initiation of renal disease, but *in-situ* generated or circulating immune complex (IC) deposits in the glomeruli are the most plausible culprits. This leads to progressive glomerular pathology and secretion of chemokines, cytokines and matrix proteins, resulting in immune cell infiltration and tissue damage. Loss of glomerular permeability also leads to tubulointerstitial injury which is perpetuated by intrinsic tubular cell inflammatory phenotype and infiltrating immune cells which eventually leads to renal failure.

Systemic lupus erythematosus (SLE) is an autoimmune disease of unknown etiology that mainly affects women of reproductive age. Lupus nephritis (LN) is the most common end-organ manifestation of SLE, affecting up to 40% of adults and 80% of children with SLE and it is a major cause of morbidity and mortality (1, 2). LN is thought to be initiated by the deposition of immune complexes, composed of anti-nuclear, anti-C1q, and cross-reactive anti-glomerular autoantibodies (3–6), in the glomeruli (7, 8). Following immune complex deposition, locally produced inflammatory mediators recruit leukocytes to perpetuate renal injury (9–11). The T and B lymphocytes from LN kidneys are clonally expanded, and the same T cell clones have been detected in the peripheral blood (12–14). A significant proportion of B cells isolated from human LN biopsies recognize vimentin, an intracellular structural protein that is cleaved and extruded from apoptotic cells (14). Autoantibodies to annexin-1 and α-enolase have also been detected in kidneys of LN patients (15). Macrophage infiltration is also a common finding in LN kidneys and is associated with poor outcomes (16–19). These intrarenal innate and adaptive immune responses may synergize with systemic autoimmunity and worsen overall outcomes (Figure 1). The combination of glucorticosteroids and cytotoxic agents, the so-called “NIH regimen,” has been the standard of care for treatment of proliferative LN for decades (20), but it is associated with significant toxicity, and results in remission in less than half of patients (21, 22). While most of the literature indicates that LN is initiated in the glomeruli, analysis of human LN biopsies indicates that the extent of tubulointerstitial lesions may better predict renal outcome (23). Below we discuss the role of tubulointerstitial injury in the pathogenesis of LN and raise the underexplored question on the role of “iron” in worsening the outcomes.

## Tubular Injury in Lupus Nephritis

A constant feature of LN is the concomitant presence of tubulointerstitial inflammation (24, 25). Tubulointerstitial inflammation, fibrosis and tubular atrophy strongly correlate with poor renal outcomes independent of the extent of glomerular damage (26). The enhanced glomerular permeability due to glomerular injury leads to overabsorption of proteins by proximal tubular epithelial cells (PTEC), triggering tubulointerstitial inflammation, scarring and renal function deterioration (27–29). Immune complex deposits have been detected in the tubular basement membrane in up to 70% of patients with LN, especially those with class III or IV LN, and the quantity of immune complex deposition correlates with the severity of tubulointerstitial inflammation (12, 29). Tubulointerstitial inflammation may be less amenable to current immunosuppressive treatment compared with glomerular proliferative changes (29). Although human PTEC express the functional glucocorticoid receptor (GRα), cytokine-induced NFkB-activation in these cells is not inhibited by glucocorticoids (30). Similarly, IL-15 production (31) and ICAM-1 expression in the kidneys and PTEC of lupus-prone MRL/lpr mice (32) were not attenuated by dexamethasone. Where glucocorticoid therapy fails to act on PTEC, *in-vitro* mycophenolic acid (MPA), the active metabolite of mycophenolate mofetil (MMF), inhibited anti-dsDNA antibody binding to PTEC and reduced the secretion of cytokines (33). However, the same study also demonstrated that continuous availability of MPA is required to sustain its anti-inflammatory actions. While MMF is a standard induction therapy for active LN, it can increase the risk of infections and cancer (34). Overall, this suggests that tubular injury in LN may proceed independently of glucocorticoid-induced immune suppression strategies (35) and the right dosage and regimen of other immunosuppressants is mandated.

Proximal tubular cells are susceptible to injury by autoantibodies, including anti-dsDNA antibodies, which adds a unique level of complexity in LN that is different than other forms of glomerulopathies. When exposed to an identical anti-dsDNA IgG concentration, a human PTEC cell line (HK-2) secreted more IL-6 than mesangial cells (29). Exposure of human PTEC to IgG and anti-dsDNA antibodies increased cellular hydrogen peroxide, activating the ROS-sensitive transcription factors NF-kB (29), ERK, MAPK, and the downstream JNK signaling pathways. This resulted in IL-6, CXCL8, CCL2, and soluble fibronectin secretion and downstream increase in profibrotic TGF-β1 and collagen synthesis (33, 36). Injured PTEC gain an inflammatory phenotype that drives the immune response by producing inflammatory cytokines including CSF-1, CCL2, IL-6, TNF, IL-1β, IL-18, directly in an autocrine manner as reviewed by Liu et al. (37), or indirectly through the production of chemokines like CCL2, CCL5, CXCL8 that recruit immune cells and worsen outcomes (38, 39). Thus, the injured PTEC are a source of chemokines and cytokines and are insensitive to glucocorticoid therapy.

The PTEC’s are also highly polar and metabolically active cells compared to other renal cells. They reabsorb 80% of the glomerular filtrate, including glucose, ions, and nutrients and, as such, they contain more mitochondria than any other cells in the kidney (40). Mitochondria, the main source of reactive oxygen species (ROS) (41, 42) produce highly reactive and toxic hydroxyl radicals (OH) via metal-dependent breakdown using cellular transition metals, most notably iron (41, 43). A redox imbalance is observed in patients with active LN and it is thought to be involved in lipid peroxidation of the glomerular basal membrane, which impairs the renal tubular function (44). High ROS levels were observed in the serum of patients with complete or partial clinical renal remission and in 92% of patients with active LN (45); moreover, multiple animal studies on LN have shown the benefit of reducing ROS (46–49).

Iron has a central role in the generation of ROS in biological systems via Fenton chemistry (50). Free Fe^2+^ ions react with hydrogen peroxide in Fenton chemistry, resulting in uncontrolled production of oxygen radicals: Fe^2+^ + H_2_O_2_ → Fe^3+^ + HO^•^ + OH^−^ then Fe^3+^ + H_2_O_2_ → Fe^2+^ + HOO + H^+^, and this reaction can worsen the outcomes of kidney injury (51). Hence iron metabolism is tightly regulated at systemic and tissue level by different check points and are discussed below.

## Systemic Iron Handling

Iron is one of the primary essential elements for life. An average human male under normal physiological conditions contains ~4 g of iron, the majority of which is distributed amongst the red blood cells and transferrin complex (Tf-Fe^3+^) in the plasma (52). Inside cells, most of the iron is complexed to ferritin, or is present in heme prosthetic or iron-sulfur groups. A small pool of chelatable iron, referred to as the labile iron pool (LIP), can take part in ROS generation, the amount of which varies between different cell types (53).

Iron is incorporated and exerts its physiological actions mainly through the iron–sulfur (Fe-S) clusters in proteins and in heme. Ferric iron (Fe^3+^) is incorporated into the Fe–S clusters within the mitochondria and subsequently incorporated into Fe–S proteins (54). Heme is produced in the mitochondria by a series of anabolic processes, and involves the integration of ferrous iron (Fe^2+^) into the center of the protoporphyrin IX ring (55). As components of the electron transport chain complexes, both Fe–S clusters and heme are involved in energy production by oxidative phosphorylation (56). Heme is also incorporated into hemoglobin and myoglobin that transport oxygen in red blood cells and muscle cells (57), and store the majority of the body's iron. Functions of Fe-S-containing proteins include ribosome modulation, transfer RNA thiolation, catalyzation the tricarboxylic acid cycle, and regulation of intracellular iron metabolism (58–61). Hence, cells maintain a sufficient supply of iron for iron-dependent processes, while at the same time restricting the size of labile iron pool to prevent excessive ROS generation from Fenton-type reactions (62).

At cellular level, iron homeostasis is a synchronized choreography of (1) uptake controlled by the transferrin receptor 1 (TfR1) and divalent metal transporter-1 (DMT-1), (2) storage by ferritin, (3) utilization in heme synthesis by erythroid 5-aminolevulinic acid synthase (Alas2); (4) and iron export by ferroportin (63–66). These processes are in turn orchestrated through the activity of iron regulatory proteins, IRP1 and IRP2, the activity of which is exquisitely sensitive to iron levels. High intracellular iron reduces the RNA-binding ability of IRP1 and the stability of IRP2. During iron deprivation, the binding of IRP1 and IRP2 to iron regulatory elements (IREs) at the 5′UTR represses the translation of Ferroportin, L-ferritin, H-ferritin, Alas2, resulting in decreased iron utilization and export, while IRP1 and IRP2 binding at the 3′UTR stabilizes *TfR1* and *DMT1* transcripts, increasing iron uptake (64, 67, 68). This, in turn, favors iron uptake over utilization and export.

**Figure 2:**
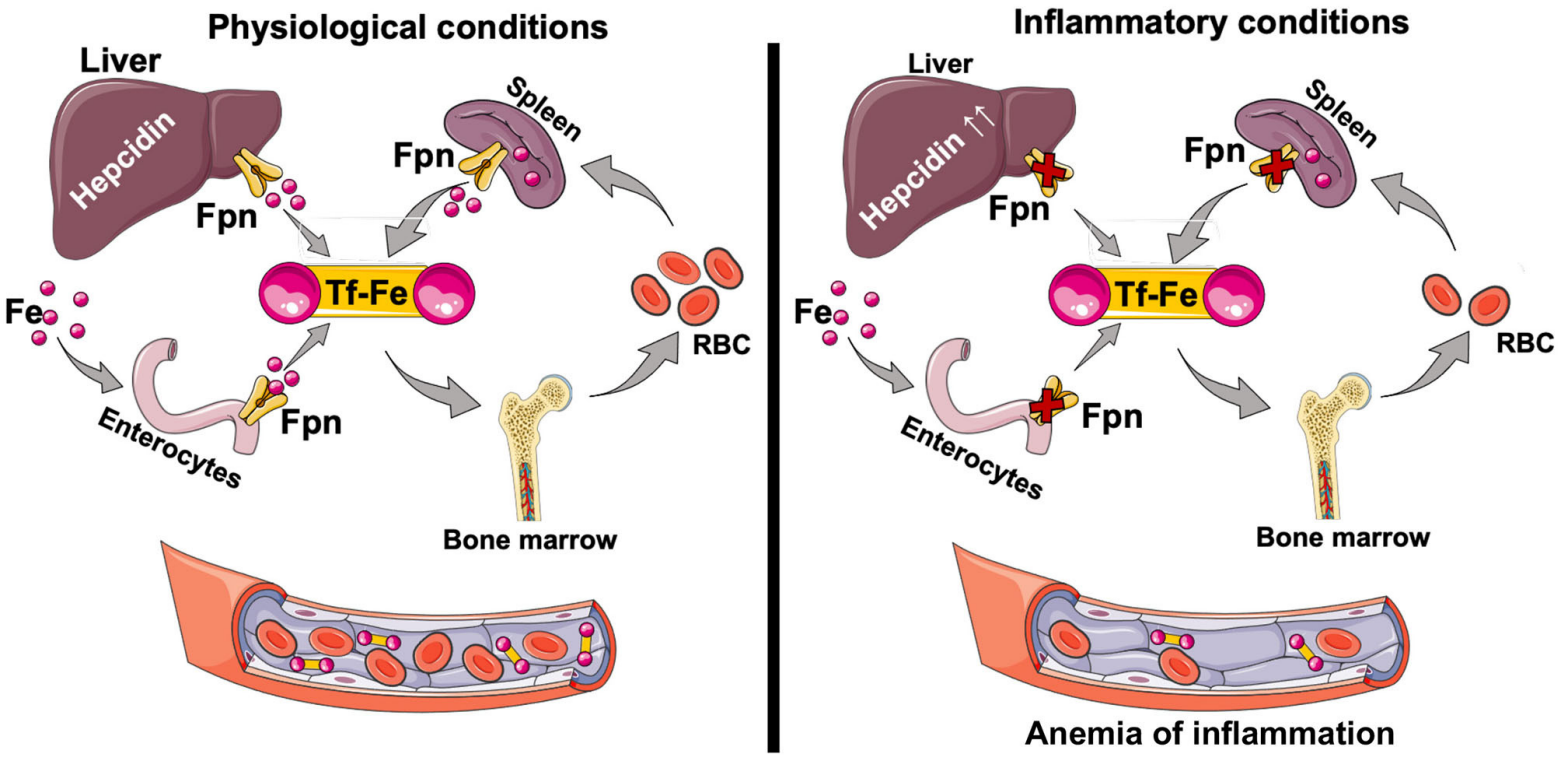
Iron absorption and circulation. Dietary iron (Fe^2+^ and Fe^3+^) is taken up through the apical surface of the duodenal enterocytes. Within the cell, iron is used for physiological purposes, or stored complexed to ferritin. Depending on the body's requirements, iron is transported through the basolateral surface of the enterocyte via ferroportin (Fpn) into circulation as the TfR-Fe^3+^ complex. The majority of the iron is used for erythropoiesis in the bone marrow. Senescent red blood cells are cleared by the splenic red pulp macrophages, which recycle iron that then can be exported for physiological functions. Changes in circulating iron levels are sensed by the liver, which produces hepcidin, a key regulator of iron homeostasis. Hepcidin-mediated FPN degradation results in iron sequestration in macrophages and net iron absorption is decreased. The net overall effect is a decrease in circulating iron levels and decreased transferrin saturation. Hepcidin is also induced by inflammation (IL-6, IL-22, Type I interferons) resulting in anemia of inflammation. Tf, transferrin; Tf-Fe, transferrin bound iron; Fpn, ferroportin.

Duodenal enterocytes are the major site of dietary iron uptake (69, 70), whereas the reticuloendothelial macrophages recycle iron from senescent RBC to collectively maintain adequate supply of iron for key physiological and developmental processes (71, 72). Systemic iron homeostasis is governed by the hepcidin-ferroportin axis (66). Hepcidin, a liver-produced hormone, regulates steady-state iron levels by binding to cell surface ferroportin, leading to its internalization and degradation. Ferroportin is the only known mammalian iron export protein that releases cellular iron into the circulation (66, 73). Genetic mutations that inhibit hepcidin production or its binding to ferroportin are associated with systemic iron overload and hemochromatosis (74, 75), while mutations in hepcidin suppressors, such as matriptase-2, cause iron refractory iron deficiency anemia (IRIDA) (76, 77). Hepcidin production is reduced by the endocrine action of erythroferrone derived from the stimulated erythroid cells (78, 79), whereas its production is stimulated by inflammation, resulting in anemia of inflammation (previously known as anemia of chronic disease) (80–82). Hepcidin-induced accumulation of intracellular iron can be toxic to the cells unless the iron is quickly and safely complexed by intracellular ferritin (83). Heavy chain ferritin (FtH), in particular, both oxidizes Fe^2+^ to Fe^3+^, rendering it non-reactive, and stores iron (43). FtH also inhibits inflammation (84–87) (Figure 2). We have shown that hepcidin can mitigate systemic inflammation in settings of renal ischemia reperfusion injury (88), endotoxemia, polymicrobial sepsis (89), and SLE (90). While systemic iron metabolism is regulated mainly by the hepcidin-ferroportin axis, at renal cell level iron metabolism is post-transcriptionally regulated mainly by the IRP-IRE system (68).

## Renal Iron Handling

Renal iron metabolism is a complex process involving iron import, storage and export and have been recently reviewed by Van Swelm et al. (91). Little is known about iron handling by the glomerular cells. Podocytes (specialized glomerular epithelial cells) can take up hemoglobin and transferrin via the megalin-cubilin complex mediated endocytosis and store the taken-up iron in ferritin (92–94). Iron can directly activate the NLRP3 inflammasome in monocytes (95), and the NLRP3 inflammasome is activated in podocytes from lupus-prone mice and from LN patients (96). The contribution of hemoglobin and transferrin bound iron in exacerbating the NLRP3 inflammasome pathway in nephritic podocytes is unknown and warrants new investigations. Cultured human glomerular endothelial cells (HGEC) express functional TfR1 and Dmt1 (iron import) and ferroportin (iron export) proteins (97). Stimulation of HGEC with both angiotensin II and apo-transferrin (iron containing transferrin) increased their labile iron content and protein oxidation products. Mesangial cells, the extracellular matrix secreting and phagocytic glomerular resident cells, express heme-oxygenase-1 (HO-1) (98) and FtH (99), and their FtH content was critical in mitigating iron toxicity and mediating the protective effect of HO-1 in response to experimental glomerulonephritis (99). Treatment of primary MRL/lpr mouse mesangial cells with NGAL (iron carrier protein) led to DNA fragmentation and cell death (100). In a rat model of experimental glomerulonephritis, hemin (oxidized heme) injection induced HO-1 and mitigated nitric oxide mediated glomerular pathology (101). It was speculated in this study that HO-1 catalyzed release of free iron can induce the cytoprotective protein ferritin, though no such evidence was provided.

Glomerular injury is a cardinal feature of LN. Within the glomeruli, podocytes, mesangial cells, and endothelial cells engage in tri-directional cross talk and influence each other as well as immune cells (102). For example, injury to the podocytes can induce mesangial cell proliferation, and mesangial cell injury can lead to foot process effacement and fusion. Signals from mesangial and endothelial cells are necessary for normal podocyte function (103). Similarly endothelin-1 from podocytes binds the endothelin-1 receptor A expressed by the adjacent endothelial cells and induces oxidative stress and endothelial cell dysfunction (104). The endothelial cell derived platelet-derived growth factor-β (PDGF-β) interacts with its receptor on mesangial cells and is critical for mesangial cell survival (105). Since all three resident glomerular cells have an active iron import, storage and export machinery, whether an intrinsic defect in or dysregulation of iron metabolism of these cells affects this self-supporting structure and outcomes of GN represents a knowledge gap that is relevant to multiple glomerulopathies.

The majority of our understanding of renal iron handling is based on studies on the tubular compartment (91, 106). The distal renal tubules express proteins associated with iron import, such as ZIP8, ZIP14, DMT1, but lack the expression of iron storage proteins (light and heavy chain ferritin) and iron export protein (ferroportin) (107). This can potentially explain iron accumulation in the distal nephron in some of the glomerulopathies like focal segmental glomerulosclerosis, diabetic nephropathy, LN, IgA nephropathy (107). Unlike the distal renal tubules, the PTEC express ZIP8, ZIP14 and DMT1 for iron import, light and heavy chain ferritin (iron storage) and ferroportin to export iron (107) and have higher abundance of IRP1 and FtH (108). Hence the distal renal tubules are unlikely to participate in iron recycling and this role is played by the PTEC (107). Under physiological conditions, a fraction of transferrin-bound iron (TBI) is filtered by the glomerulus into the renal tubular lumen and is then almost completely reabsorbed by renal tubular epithelial cells (109, 110). TBI is imported from the apical surface of the PTEC via TFR1 and megalin-cubulin endocytic complex (111, 112), whereas non-transferrin bound iron (NTBI) is imported by ZIP8 and/or ZIP14 (107, 113), such that iron loss in the urine is minimal. Megalin is also known to take up hepcidin (114), but the consequence of this interaction is not known. PTEC express FtH which can sequester and oxidize Fe^2+^ and ferroportin to export some of the reabsorbed iron on the basolateral side (91, 107). Loss of FtH from PTEC sensitizes them to both acute kidney injury (115) and fibrosis (116). Since dietary sources of iron were scarce during our evolutionary past, it is likely that there was a selection pressure for multicellular organisms to minimize iron loss. This may explain why PTEC are endowed with proteins that import iron from luminal surface, store it safely intracellularly and export it basolateral to minimize urinary iron loss.

The combination of filtered iron uptake and a high mitochondrial content render the PTEC susceptible to iron-catalyzed, ROS-mediated injury. Hence, IRP1 is highly abundant in the PTEC (91, 117) and regulates their iron content by synchronizing TfR1, DMT1 (uptake), FtH (for storage), and ferroportin (for export).

## Iron and The Pathogenesis of Autoimmune Disease

Little is known about the mechanistic role of iron in autoimmune disorders such as rheumatoid arthritis (RA) and SLE and associated pathologies. Patients with RA have higher concentrations of free iron and other iron-binding proteins in synovial fluid relative to those without the disease (118, 119). Iron-dextran infusion exacerbated rheumatoid synovitis by increasing lipid peroxidation, oxidized ascorbic acid and by decreasing red cell glutathione (120, 121), thus directly implicating iron in the pathogenesis of RA and rheumatoid synovitis. Dietary iron aggravates human lupus (122) and iron infusion worsens the disease activity (123). Urine ferritin and Tf levels are elevated in SLE patients and correlate with disease activity (124). Neutrophil gelatinase-associated lipocalin (NGAL), one of the most highly up-regulated proteins in acute kidney injury, is an iron carrier protein that predict the course of global and renal childhood-onset SLE disease activity (125). Akin to humans, lupus-prone female MRL/lpr mice fed with iron-supplemented or severely iron-deficient diets had higher mortality than those with moderate iron deficiency, or control diet (126). This early study highlighted that in SLE dysregulation of iron metabolism is associated with end organ pathology, whereas the commonly used autoimmune biomarker like anti-dsDNA antibody level is not affected.

## Iron in Lupus Nephritis

In patients with chronic kidney disease, iron accumulates in the lysosomes (site of iron processing but not storage) of damaged, but not undamaged, PTEC (127). Renal biopsies of primary glomerulopathies such as focal segmental glomerulosclerosis, anti-glomerular basement membrane disease and IgA nephropathy, as well as secondary ones such as LN, show iron deposits in PTEC and distal tubular cells, and also stain positive for oxidative stress-induced protein heme oxygenase-1 (107). Recently, in a study involving 120 SLE patients, transferrin and ceruloplasmin (ferroxidase) (128) were proposed as potential biomarkers for LN (129). This study confirmed the previous observations by Suzuki et al. which demonstrated that urinary transferrin and ceruloplasmin were significantly higher with active vs. inactive LN or in SLE patients without renal involvement (130). The same authors also found that urinary NGAL (iron carrier protein) could represent a novel biomarker for renal disease activity in pediatric SLE (131). Along similar lines, urinary transferrin was found to be a positive predictor of future renal functional decline in pediatric and adult LN (132). In another human study, Indrakanti et al. (133) found that during renal and non-renal SLE flares, IL-6 did not correlate with hepcidin and hepcidin did not predict hemoglobin. However, when LN patients were in remission, IL-6 and hepcidin were correlated, but hepcidin and hemoglobin did not. The authors thus concluded that hepcidin does not contribute significantly to anemia during active lupus (133). Using low molecular weight proteome to predict impending renal relapse, relapse severity, and the potential for recovery after SLE nephritis flare, it was observed that hepcidin 20 (isoform of hepcidin that lacks the first five amino acids of the amino-terminal portion) increased 4 months before renal flare and returned to baseline during renal flare, whereas hepcidin 25 decreased during renal flare and returned to baseline 4 months after the flare (134). However, unlike the observations in RA (120, 121) and SLE (122, 123), where dietary iron or iron infusion worsened disease activity, studies on role of iron in worsening LN are correlational and not causal. Tubular iron accumulation is also a feature in animal models of LN (135–137). In an induced model of glomerulonephritis caused by injection of nephrotoxic serum (138), phlebotomy mitigated tubulointerstitial disease and renal functional deterioration though the glomerular injury was comparable to non-phlebotomized rats (135). The reduction in iron content caused by the phlebotomy correlated inversely to functional deterioration and extent of tubulo-interstitial disease.

Only two animal studies have investigated the pharmacological modulation of iron metabolism to mitigate LN. The PTEC of proteinuric NZBWF1 mice, a well-established spontaneous model of SLE/LN showed a lower expression of transferrin receptor 1 (TfR1: iron importer) and increased expression of ferritin, indicative of iron accumulation (136). Treating these mice with deferiprone, an FDA-approved iron chelator, delayed the onset of albuminuria even though anti-dsDNA IgG levels were similar to the vehicle treated group (136). Furthermore, markers of tubular injury and renal function were significantly lower in the deferiprone-treated mice.

We recently reported that regulation of iron metabolism using exogenous hepcidin reduced renal iron accumulation, labile iron content, and injury parameters in MRL/lpr mice, another spontaneous model of SLE/LN (90). As in the NZBWF1 mice, regulation of iron metabolism with hepcidin treatment did not reduce renal immune complex deposits and serum autoantibodies, but it mitigated intrarenal cytokine production, immune cell infiltration and parenchymal injury, including tubular injury, without worsening lupus associated anemia. Importantly, hepcidin was protective even when administered to proteinuric mice, highlighting its therapeutic potential.

Our observations are counter intuitive to the current paradigm that suggests that inhibition of ferroportin-induced iron export should worsen iron-mediated injury. We observed that, intermittent administration of exogenous hepcidin more than doubled the expression of renal FtH (90), a cytoprotective molecule. In support of our observation, in a rodent model of thymocyte antigen-1-induced glomerulonephritis, heme oxygenase-1 (HO-1) blockade lowered the expression of FtH and accelerated mesangial cell death (99). Forced expression of wild-type FtH overcame HO-1 deficiency and made the cells more resistant to ROS-mediated injury and this salutary effect was not observed in FtH mutants that lost the capacity of iron storage and ferroxidase activity. The importance of PTEC FtH was previously established in models of acute kidney injury (115) and unilateral ureteral obstruction (116). Moreover, FtH inhibits MAPK signaling (86), suppresses the proliferation of T cells and impairs the maturation of B cells in autoimmune diseases (84, 87).

The results from these pre-clinical studies suggest that reducing the availability of labile iron using different approaches increases the resistance of renal parenchymal cells to SLE-associated insults, especially since circulating autoantibodies and glomerular immune complex deposits were not affected by the treatment.

## IRP-IRE Independent Iron Uptake: A Potential Link to Iron-Mediated PTEC Injury

Increased filtration of albumin causes excessive tubular reabsorption, resulting in inflammation and fibrosis, which is thought to be a major contributor to tubulointerstitial injury (139). However, injury to the glomerular structure results in increased permeability to all proteins, including apo-transferrin. Since the PTEC reabsorb the majority of filtered iron, under pathological conditions these cells are more susceptible to iron-mediated oxidative injury. The PTEC express TfR1, megalin-cubulin complex as well as Zip8 and 14 on their apical surface. Unlike TfR1, the receptor for transferrin, which is post-transcriptionally regulated by the IRP-IRE system and is downregulated in cells that accumulate excess iron (140), the expression of megalin-cubulin endocytic complex is not regulated by cellular iron content. Similarly, ZIP8 and ZIP14 are both involved in NTBI uptake, and ZIP14 also mediates TBI-derived iron uptake (113), and their abundance is not regulated by IRE-IRP system (141). Thus, following the breakdown of glomerular filtration barrier, the TBI can be taken up by the PTEC in disproportionate manner, dissociate from Tf in the highly acidic environment of the lysosome (142) and accumulate as cytoplasmic pool of labile iron.

**Figure 3:**
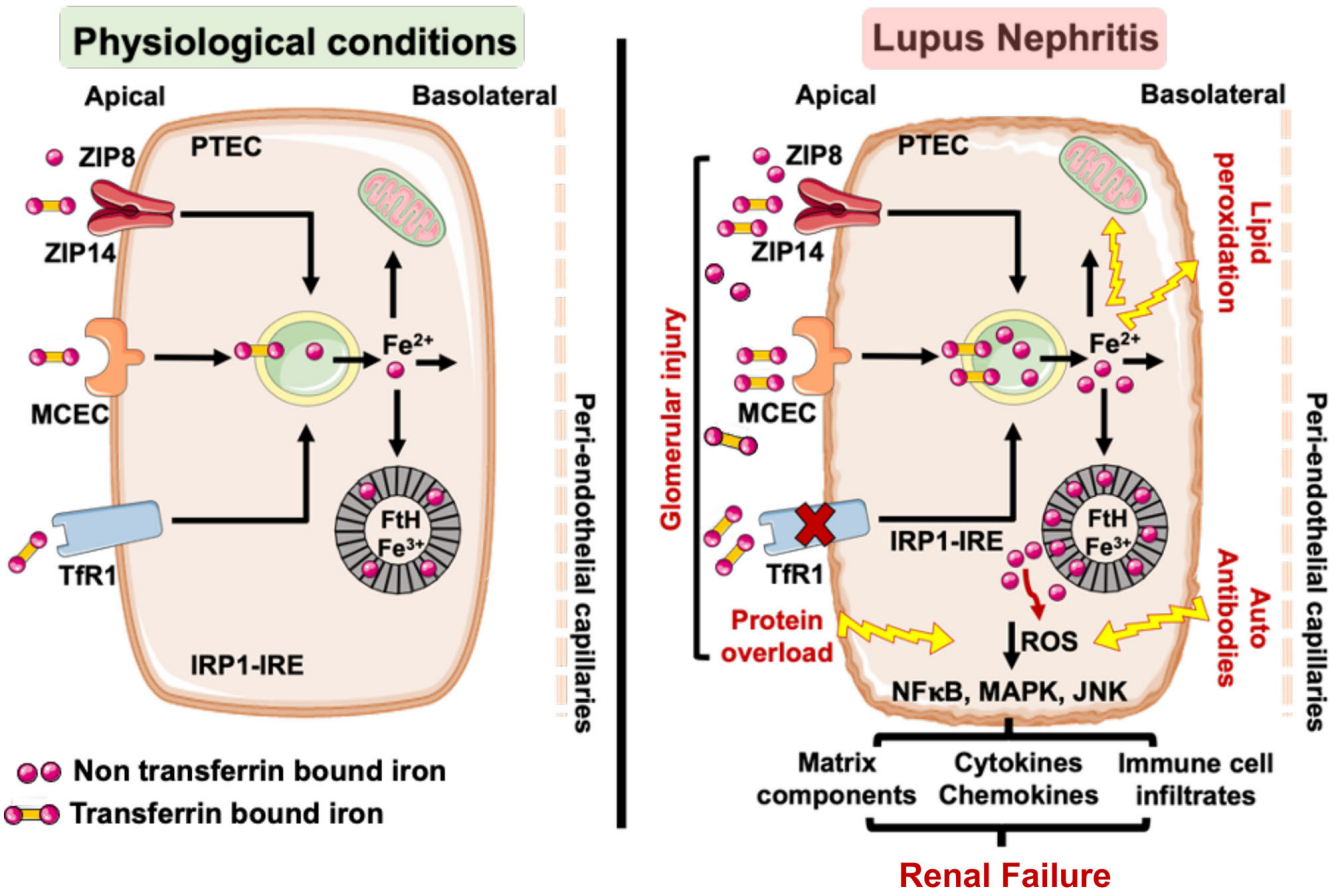
Under physiological conditions little transferrin bound (TBI) and non-transferrin bound iron (NTBI) is filtered by the glomerular assembly and is reabsorbed and cycled by the proximal tubular cells (PTEC). However, in LN, glomerular injury results in an increased leakage of TBI and NTBI, which can be reabsorbed by the PTEC via TfR1, ZIP8/14, and MCEC. While TfR1 is regulated by the IRP-IRE system, ZIP8/14, and MCEC are not and can continue to absorb the leaking TBI and NTBI to iron overload the PTEC. This can overwhelm the heavy chain ferritin (FtH) iron binding capacity, leading to release of labile iron and render the PTEC susceptible to ROS mediated injury and lipid peroxidation. The glomerulopathy associated protein overload (e.g., albumin) and lupus autoantibodies can independently induce ROS in the PTEC and activate ROS sensitive inflammatory pathways. The accumulated iron can catalyze ROS formation via the Fenton reaction and exacerbate the inflammatory phenotype of the PTEC to worsen tubulointerstitial injury and accelerate the progression to renal failure. TfR1, transferrin receptor 1; MCEC, megalin cubulin endocytic complex; FtH, heavy chain ferritin; ROS, reactive oxygen species; IRP-IRE, iron regulatory protein-iron response element.

Collectively, these observations are consistent with a model (Figure 3) wherein the loss of glomerular perm-selectivity in LN leads to enhanced uptake of TBI and NTBI by the PTECs via megalin-cubulin endocytic complex, ZIP14 and ZIP8 in a TfR1 independent manner, leading to a chronic increase in intracellular labile iron (Fe^2+^). Excess intracellular iron is expected to increase the synthesis of IRE-IRP regulated FtH (67), which can sequester the labile iron. But continued glomerular leakiness can overwhelm this defense mechanism. LN is also associated with lower levels of antioxidant (45, 143, 144) and collectively lead to oxidative damage and lipid peroxidation. Lipid peroxidation, a striking feature of ferroptosis (regulated cell death characterized by the iron-dependent accumulation of lipid hydroperoxides) (145–148) is increased in both SLE patients (149, 150) and mice (151). Excess labile iron also catalyzes the formation of ROS (62, 152). IgG and anti-dsDNA antibodies in lupus patients induce ROS, which upregulates multiple inflammatory pathways in PTEC (29, 33, 36). By catalyzing the formation of ROS (62, 152), labile iron may accentuate PTEC inflammatory response to IgG and anti-dsDNA antibodies. Thus, iron-induced lipid peroxidation and exacerbation of inflammatory responses can synergistically accelerate tubulointerstitial injury and progression to renal failure. Alternatively, the accompanying albuminuria in LN and other glomerulopathies is known to induce mitochondrial ROS-mediated activation of the cytoplasmic NLRP3 inflammasome pathway in PTEC (153) and stimulate proximal tubular cells to synthesize chemokines that recruit immune cells. While iron can exacerbate ROS production (152) and cause mitochondrial damage (154), it can also directly activate the NLRP3 inflammasome in monocytes, the other iron handling cells (95). The NLRP3 inflammasome is an attractive target to treat LN (155) and its expression in glomerulonephritis remains largely confined to the tubules (156), a site of iron loading in the kidney. While glomerular cells are affected by iron (98–101), both human and animal studies demonstrate visible iron deposits in the tubular segments. As the PTEC's are the major iron handing cells in the kidney, iron affects the tubular segment more profoundly than the glomerular cells in settings of LN. However, no study has compared iron induced injury in glomerular and tubular cells in settings of LN and requires more investigations.

## Future Questions and Implications

The finding that the renal iron accumulation is associated with increased injury and inflammation in LN poses a number of pertinent questions. While this phenomenon is now well-documented by multiple independent studies, the mechanisms by which iron mediates and perpetuates tubulointerstitial inflammation following glomerular injury is not yet defined. Both induced and spontaneous models of LN indicate that decreasing labile iron content using different strategies protects against renal failure in LN, independent of the cardinal autoimmune disease biomarkers, such as autoantibodies and glomerular immune complex deposits. The mechanistic role and consequence of inhibiting iron induced PTEC lipid peroxidation or ferroptosis in LN has not yet been explored and is a new frontier in this complex disease. Targeting ferroptosis could open a novel research avenue and future adjunct therapy to treat a cell that is insensitive to glucocorticoid therapy. T lymphocytes express ferroportin (157), take-up NTBI (158) and iron induces epigenetic changes in SLE patients CD4^+^ T cells (159). Whether iron causally affects B lymphocytes is unknown. This implies that a deeper understanding of renal vs. systemic iron homeostasis is necessary. Iron metabolism is an easily druggable target that could synergize with existing immunotherapies that mainly act on the immune aspects of SLE and LN. In this regard, VIT-2763 (oral ferroportin inhibitor), synthetic human hepcidin and its agonists that are currently in clinical trials (ClinicalTrials.gov: NCT04364269, NCT03381833, and NCT03165864) could be tested as adjuvants to immunosuppressive therapy for LN. A word of caution is however mandated as long-term use of these interventions could potentially worsen the anemia that is commonly associated with drugs regulating iron metabolism.

## Author Contributions

YS conceptualized and designed the article. EW and YS made the figures and wrote the manuscript. BM and LM edited and wrote sections. All authors edited and approved the final version of the manuscript.

## Conflict of Interest

The authors declare that the research was conducted in the absence of any commercial or financial relationships that could be construed as a potential conflict of interest.

## Abbreviations

LN: Lupus nephritis
Fe^2+^: labile bioactive iron
Tf: Transferrin
TfR1: Transferrin receptor 1
TBI: Transferrin bound iron
NTBI: non-Transferrin bound iron
PTEC: Proximal renal tubular cell
IRP1 and IRP2: iron regulatory protein 1 and 2
IRE: iron response elements
FtH: Heavy chain ferritin.
